# A Case Report of Postoperative Cystic Hydrocephalus Following Duroplasty and Scalp Reconstruction in Neonatal With Aplasia Cutis Congenita and Extensive Craniodural Defect

**DOI:** 10.1155/cris/7741728

**Published:** 2026-07-30

**Authors:** Agung Budi Sutiono, Betha Egih Riestiano, Yulius Hermanto, Jusuf Sulaeman Effendi, Nana Sanardi, Yani Surya Dewi

**Affiliations:** ^1^ Department of Neurosurgery, Mayapada Hospital Bandung, Bandung, West Java, Indonesia, mayapadahospital.com; ^2^ Department of Neurosurgery, Faculty of Medicine, Padjajaran University - Dr. Hasan Sadikin Hospital, Bandung, West Java, Indonesia; ^3^ Department of Plastic Surgery, Faculty of Medicine, Padjajaran University - Dr. Hasan Sadikin Hospital, Bandung, West Java, Indonesia; ^4^ Department of Neurosurgery, Santo Borromeus Hospital, Bandung, West Java, Indonesia; ^5^ Department of Obstetrics and Gynaecology, Mayapada Hospital Bandung, Bandung, West Java, Indonesia, mayapadahospital.com; ^6^ Department of Paediatrics, Mayapada Hospital Bandung, Bandung, West Java, Indonesia, mayapadahospital.com

**Keywords:** aplasia cutis congenita, case report, dural aplasia, hydrocephalus, scalp defect

## Abstract

**Background:**

Aplasia cutis congenita (ACC) is a rare congenital disorder characterized by localized absence of skin, most frequently involving the scalp. In severe cases, the defect may extend to the skull and dura mater, exposing intracranial structures and increasing the risk of infection, hemorrhage, and venous sinus injury. Although early surgical reconstruction with duraplasty and scalp flap coverage is often successful, delayed complications such as cystic hydrocephalus may develop months after surgery. Extensive ACC with both cranial and dural defects remains exceedingly rare, and optimal management strategies are not well established.

**Case Presentation:**

We report a full‐term female newborn with a large midline parietal scalp defect measuring 7 cm × 6 cm, associated with absence of the underlying calvarium and dural aplasia. The lesion was covered by a thin membranous tissue with visible brain pulsation and exposure of the superior sagittal sinus (SSS). Initial management included sterile dressings and broad‐spectrum antibiotics. On day 8 of life, surgical reconstruction was performed using synthetic duraplasty followed by rotational scalp flap closure. Postoperative recovery was uneventful, and the wound healed satisfactorily. At 6 months postoperatively, the patient developed cystic hydrocephalus, which was managed with ventriculoperitoneal shunt placement. At 24‐month follow‐up, the patient achieved normal developmental milestones with a good cosmetic outcome.

**Conclusion:**

Extensive ACC involving the skull and dura mater represents a severe and potentially life‐threatening variant of the disease. Early multidisciplinary management with synthetic duraplasty and scalp flap reconstruction can provide effective protection of intracranial structures and yield favorable long‐term outcomes. Nevertheless, long‐term follow‐up is essential, as delayed complications such as cystic hydrocephalus may arise months after initial surgical repair.

## 1. Introduction

Aplasia cutis congenita (ACC) is a rare congenital anomaly characterized by the localized absence of skin, with an estimated incidence of approximately 1 in 10,000 live births [1, 2]. The vertex scalp is the most frequently affected site, accounting for 70%–90% of cases [1, 2]. Lesions exhibit wide variability in size and depth, ranging from small superficial skin defects to extensive areas involving the absence of the underlying skull and dura mater; approximately 20% of scalp ACC cases are associated with bony defects [1–3].

Most superficial lesions heal spontaneously by secondary intention; large midline defects with exposure of the dura or brain tissue require urgent surgical intervention due to the high risks of hemorrhage, meningitis, and desiccation [1–4]. The reported mortality associated with large ACC is around 10% [1].

The pathogenesis of ACC remains incompletely understood and is thought to be multifactorial. Proposed mechanisms include genetic predisposition, teratogenic exposure (such as methimazole or misoprostol), intrauterine infections, vascular compromise leading to localized ischemia, amniotic band disruption, and mechanical or traumatic factors during gestation [1, 3, 5, 6]. These diverse etiologies converge on a shared developmental failure of the ectodermal and mesodermal layers, resulting in the localized absence of skin and associated structures, which explains the marked variability in clinical presentation and severity [1, 3–7].

In this report, we describe a rare case of extensive scalp ACC associated with a large cranial vault and dural defects in a neonate, successfully managed with synthetic duraplasty and rotational scalp flap reconstruction. We also review similar cases in the literature and discuss the potential for delayed complications, including cystic hydrocephalus.

## 2. Case Presentation

**Figure 1:**
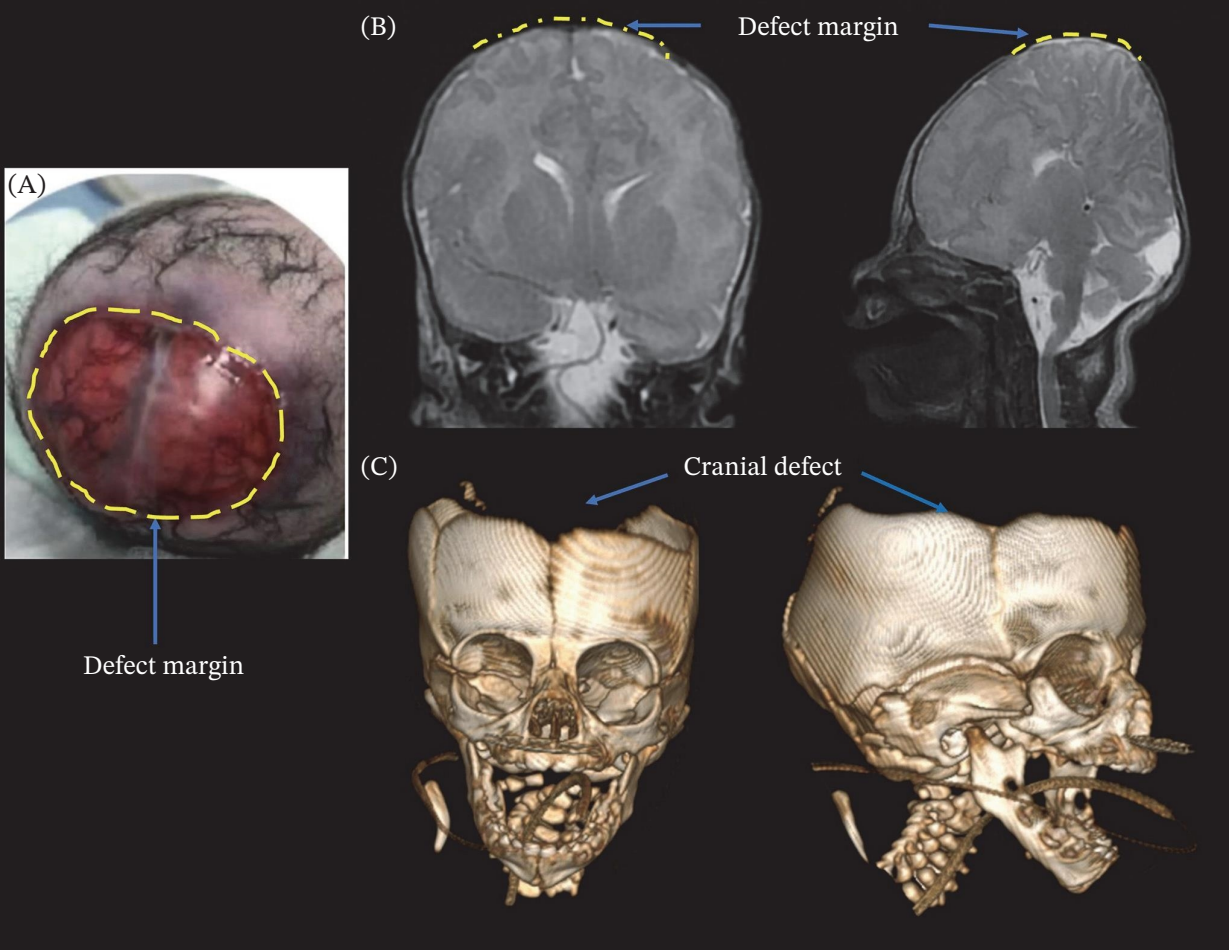
Patient’s preoperative condition. (A) clinical picture of a large defect of aplasia cutis congenita involving the skull and dura mater. (B) Head MRI reveals normal intracranial content without hydrocephalus or other congenital anomalies. (C) Head CT‐scan reveals a large skull defect involving the parietal bones.

A full‐term female neonate was born to a healthy, nonconsanguineous mother after an uneventful pregnancy. The birth weight was 3.2 kg with normal Apgar scores. At birth, a well‐demarcated, oval‐shaped defect measuring 7 cm × 6 cm was observed over the vertex and midline parietal regions. The area was devoid of skin and subcutaneous tissue, revealing a thin, translucent membrane with visible brain pulsation. The underlying skull bone was absent, and part of the dura mater was deficient, with exposure of the superior sagittal sinus (SSS) (Figure 1A).

No other congenital anomalies, limb defects, or neurological abnormalities were detected. Head magnetic resonance imaging (MRI; Figure 1B) and CT‐scan (Figure 1C) confirmed a large skin and skull defect involving the parietal bones, without evidence of encephalocele or intracranial malformation.

The neonate was admitted to the neonatal intensive care unit for monitoring. Initial management included sterile nonadherent dressings with paraffin gauze, prophylactic intravenous antibiotics, and maintenance of normothermia and hydration.

Due to the inherent risks of infection and sagittal sinus injury, surgical repair was performed on the eighth day of life. Under general anesthesia, the neonate was positioned supine with the head slightly elevated to reduce venous pressure. The thin membranous covering over the defect was meticulously excised, exposing the underlying neural tissue and SSS. The sinus was noted to be intact but superficially located, with no overlying dural protection. To avoid sinus laceration or thrombosis, several precautions were taken: no electrocautery was used directly on or near the sinus; sharp dissection with micro‐scissors was employed to free adherent arachnoid adhesions; application of topical hemostatic agents at the field; and gentle irrigation with warm saline was used to maintain a moist environment and prevent desiccation of the sinus wall.

Dural reconstruction was then performed. A synthetic collagen‐based dural substitute was selected over autologous grafts for the following reasons: the patient’s limited autologous tissue availability, as the large scalp defect lacked pericranium and the temporalis muscles were underdeveloped in a neonate; the need for a watertight, nonshrinkable barrier to prevent cerebrospinal fluid (CSF) leakage and promote neomembrane formation; the synthetic substitute provided a smooth, nonadherent surface over the exposed sinus and brain parenchyma, minimizing the risk of intraoperative sinus injury during graft placement; it avoided donor site morbidity in a critically ill neonate; and synthetic duraplasty allowed for a standardized, readily available implant without prolonged operative time for autologous graft harvest. The graft was trimmed to size and sutured circumferentially to the dural edges using interrupted 6–0 polypropylene sutures, taking care not to compress the SSS.

**Figure 2:**
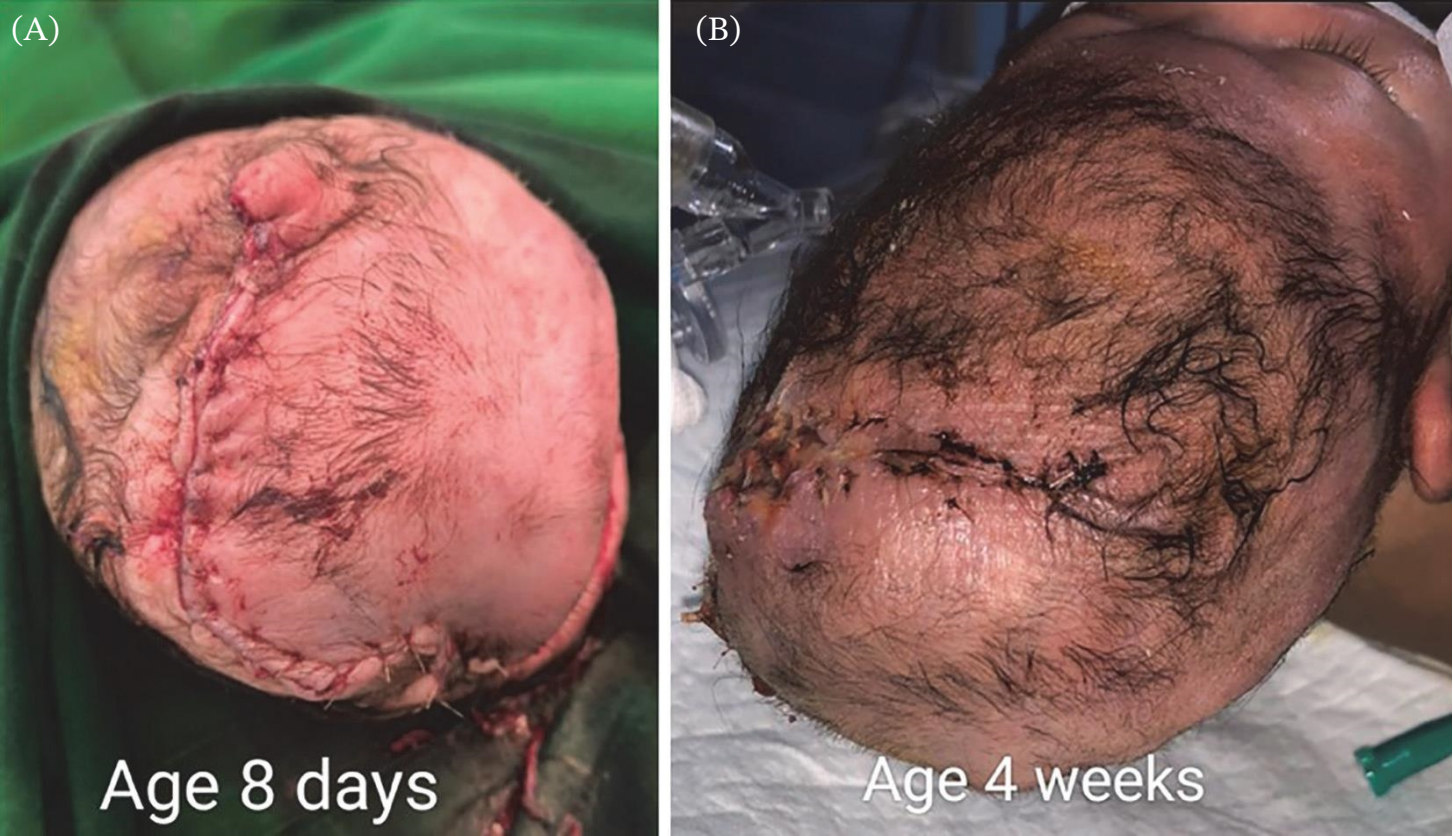
Postoperative clinical images. (A) On day 8 of life, the patient underwent surgical reconstruction consisting of synthetic duraplasty and a rotational scalp flap for defect closure. (B) At 4 weeks postoperatively, the wound demonstrated complete healing with good flap viability and no evidence of infection or CSF leakage.

The overlying scalp defect was reconstructed using bilateral rotational scalp flaps, elevated in the subgaleal plane, advanced medially, and sutured in two layers (galea and skin) to achieve tension‐free closure (Figure 2A). A closed suction drain was placed in the subgaleal space for 48 h. The postoperative period was uneventful. The infant was discharged after 4 weeks with complete flap viability (Figure 2B).

**Figure 3:**
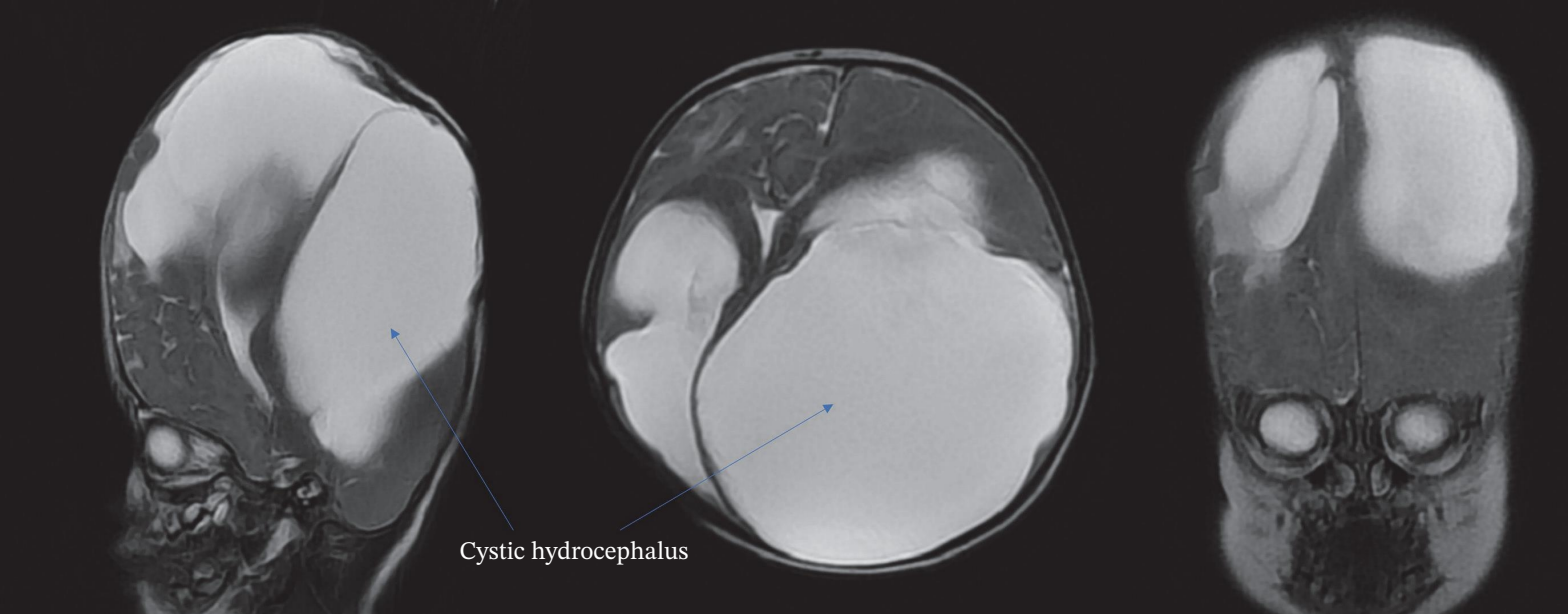
Follow‐up MRI at 6 months postoperatively. 6 months after dural reconstruction and scalp flap repair, the patient developed cystic hydrocephalus.

At the 6‐month follow‐up, the wound was well healed; however, the patient’s head circumference had increased beyond the normal range for her age. Follow‐up MRI demonstrated cystic dilatation of both lateral ventricles, consistent with hydrocephalus (Figure 3). She subsequently underwent ventriculoperitoneal shunt placement. She was routinely followed thereafter. At 24 months of follow‐up, she was otherwise healthy, with normal growth and developmental milestones, without abnormality in head circumference.

## 3. Discussion

ACC presents with varying severity, and its management depends on the lesion size, depth, and associated anomalies. The majority of small defects (<2 cm) heal by secondary intention; however, larger lesions with exposed dura or brain tissue require surgical closure [1–10]. Large ACC over the vertex often involves the sagittal suture and SSS, creating challenges in surgical management due to the risks of sinus injury and massive bleeding [1–10].

The optimal timing of surgery for ACC remains a topic of debate. Most studies advocated conservative treatment to promote granulation and epithelialization of the defect [1, 2, 8, 11, 12]. However, other studies emphasize the benefits of early surgical intervention, particularly with dural aplasia, ideally within the first week of life, to reduce the risk of infection, prevent CSF leakage, and protect the exposed venous sinus and brain [1, 3–7, 13–15]. Pollock et al. [14] and Yang et al. [16] both reported successful outcomes with early operative repair using dural closure and scalp flap coverage, with minimal postoperative complications. In our case, surgical repair was performed on day 8 of life, in line with these recommendations, leading to rapid recovery and excellent wound healing.

Reconstruction of the dural defect is a crucial step in managing ACC with dural aplasia. Traditionally, autologous grafts (fascia lata or pericranium) have been preferred for their biological compatibility and lower risk of infection [17–19]. A recent meta‐analysis concluded that autografts are more effective than nonautografts for reducing postoperative meningitis, pseudomeningocele, and wound infection [19]. Additionally, a series of pediatric posterior fossa surgeries demonstrated that an autologous graft is associated with fewer complications [20].

Synthetic dural substitutes, including collagen‐based matrices, xenografts, and acellular dermal matrices, have gained acceptance due to advances in biomaterials [3–7, 21–23]. Their primary advantage is the elimination of donor‐site morbidity and reduced operative time, as no second incision is required [3–7, 21–23]. In our case, a synthetic collagen‐based dural graft was selected because the large scalp defect lacked usable pericranium and the temporalis muscles were underdeveloped, making autologous harvest impractical. The graft provided satisfactory structural integrity and prevented postoperative CSF leakage or infection, supporting its short‐term safety and efficacy in this neonate.

The management of scalp coverage depends on the defect’s size and vascularity. Small, superficial lesions may be managed conservatively or with skin grafts, while large defects often require local or rotational scalp flaps to ensure vascularized coverage [3, 4, 7, 17, 18, 24]. Local scalp flaps with or without prior tissue expansion are widely used; free flaps and muscle flaps are reserved for very large or recurrent defects where the local tissue is insufficient [3, 4, 7, 17, 18, 24]. In our patient, bilateral rotational scalp flaps achieved tension‐free closure with complete viability.

For cranial vault reconstruction, both autologous bone grafts (rib or split calvarium) and artificial bone substitutes have been described; the choice depends on the defect size and the patient’s age [1, 25–27]. Spontaneous bone formation has been observed in infants, suggesting that immediate cranioplasty may be deferred in select patients [1, 2, 8, 11, 12]. In this case, we elected to defer cranioplasty to allow for possible spontaneous ossification. We summarized the reported strategies for managing ACC in Table S1.

Although the immediate postoperative outcome in ACC is often favorable, delayed complications may occur. Hydrocephalus, though rare, is a notable late complication after dural and scalp reconstruction. The pathogenesis of hydrocephalus is hypothesized to involve impaired CSF absorption due to altered arachnoid granulation function, obstruction of venous drainage at the SSS, or fibrosis and impaired CSF egress through the reconstructed dura [7, 28, 29]. In some patients, early cystic accumulation of CSF beneath the reconstructed dura can progress to hydrocephalus [7, 28, 29]. The delayed presentation (months after surgery) observed in our patient is consistent with a gradual impairment of CSF resorption rather than acute obstruction. We successfully managed hydrocephalus with a ventriculoperitoneal shunt. This emphasizes the importance of long‐term follow‐up with neuroimaging surveillance in neonates undergoing ACC repair involving dural reconstruction.

To the best of our knowledge, this is one of the few reported cases of extensive ACC with both cranial and dural defects successfully managed in the neonatal period using synthetic duraplasty and rotational scalp flap reconstruction. The subsequent development of delayed cystic hydrocephalus 6 months postoperatively represents a rare but important complication not previously well‐documented in the literature. This case highlights the feasibility of early multidisciplinary intervention, even in resource‐limited settings, and underscores the need for long‐term neuroimaging surveillance following dural reconstruction in neonates.

## 4. Limitations

This report is a single case with a 24‐month follow‐up, which limits the generalizability of our findings. Longer‐term surveillance is required to assess potential late sequelae, such as the need for further cranial reconstruction or the long‐term function of the ventriculoperitoneal shunt. Additionally, while we describe an association between synthetic duraplasty and delayed cystic hydrocephalus, a causal relationship cannot be established from a single observation.

## 5. Conclusion

Extensive ACC involving both the cranial bone and dura represents one of the most severe variants of this rare congenital disorder. Prompt diagnosis and intervention should be tailored. In case of extensive dural involvement, a synthetic dural substitute and vascularized scalp flap reconstruction can achieve excellent structural and cosmetic results while minimizing the risk of infection and CSF leakage. However, this case highlights that delayed complications such as cystic hydrocephalus may emerge months after the initial recovery, emphasizing the importance of prolonged clinical and radiologic surveillance. Awareness of this potential sequela is crucial for timely recognition and management, ensuring optimal long‐term neurological and developmental outcomes.

## Funding

No funding was received for this study.

## Disclosure

All scientific content, interpretations, and conclusions were conceived, reviewed, and approved by the authors, who take full responsibility for the final version of the manuscript.

## Ethics Statement

The study was reviewed and approved by the Mayapada Hospital Bandung, Bandung, Indonesia (Approval Number MHB/EC/2025/022).

## Consent

Written informed consent was obtained from both parents for the publication of this case report and any accompanying clinical images, including MRI and CT scans.

## Conflicts of Interest

The authors declare no conflicts of interest.

## Supporting Information

Additional supporting information can be found online in the Supporting Information section.

## Supporting information


**Supporting Information** Table S1: List of reported literatures about aplasia cutis congenita of the scalp.

## Data Availability

The data that support the findings of this study are available upon request from the corresponding author. The data are not publicly available due to privacy or ethical restrictions.
