# Supplementary material for: A Case Report of Postoperative Cystic Hydrocephalus Following Duroplasty and Scalp Reconstruction in Neonatal With Aplasia Cutis Congenita and Extensive Craniodural Defect
Source: Case Rep Surg. 2026 Jul 30;2026:7741728. doi: 10.1155/cris/7741728 (PMC13424449; doi:10.1155/cris/7741728)
Supplement: Supplementary file 1 — Supporting Information Table S1: List of reported literatures about aplasia cutis congenita of the scalp. [file CRIS-2026-7741728-s001.docx]

|  | **Authors** | **Year** | **Chronological Age, days** | **Sex** | **Syndromic**  **(Yes/No/Not Available)** | **Co-morbidities** | **Location** | **Depth of skull defect** | **Sagittal sinus exposure (Yes /No)** | **Time to Treatment** | **Conservative treatement** | **Surgical management** | **Treatment outcome** | **Complications** | **Mortality (Yes/No)** |
| --- | --- | --- | --- | --- | --- | --- | --- | --- | --- | --- | --- | --- | --- | --- | --- |
| 1 | Agyekum R, et al.^1^ | 2026 | 1 day | Female | N | NA | Vertex | Skull and dura absent | Y | Delayed | Moist dressings and antibiotics | Yes, duroplasty with bovine pericardium | Good. | Surgical site infection | N |
| 2 | Uribe-Cavero LJ, et al.^2^ | 2026 | 2 day | Male | N | NA | Vertex | Skin absent | N | Early | Moist dressings and antibiotics | None | Good | None | N |
| 3 | Pollock M, et al.^3^ | 2025 | Newborn | NA | NA | NA | Occipital | Skin and skull bone absent | N | Early | None | Application of PolyNovo biodegradable temporising matrix (BTM) synthetic dermal substitute | Good | None | N |
| 4 | Bouali S, et al.^4^ | 2024 | Newborn | Not specified | N | NA | Vertex | Skin and bone absent | Y | Early | Moist dressings, debridement, silver-coated dressings, and antibiotics | None | Good | None | N |
| 5 | Rose MD.^5^ | 2024 | Newborn | Male infant | N | NA | Vertex | Skin and bone absent | N | Early | Moist dressings and observation | None | Good | None | N |
| 6 | Al Matrafi FR, et al.^6^ | 2023 | 23 days | Female | Trisomy 13 | Cardiac abnormalities, Orofacial cleft | Vertex | Skin and bone absent | N | None | Moist gauze dressing, topical antibiotics | None; DNR decision made | Managed conservatively, no surgical intervention | Not Applicable | Y |
| 7 | Yang XF, et al.^7^ | 2023 | Newborn | Male | Adams–Oliver syndrome | NA | Vertex | Skin and bone absent | N | Early | recombinant human EGF gel and kangfuxin solution | None | Good | None | N |
| 8 | Karakawa R, et al.^8^ | 2021 | Newborn | Male | Adams–Oliver syndrome | Skin infection | Vertex | Skin and bone absent, partial dura defect | Y | Delayed | Initially moist dressing | Dural reconstruction using fascia lata + scalp reconstruction with transposition flap | Good | None | N |
| 9 | Scotti A, et al.^9^ | 2021 | Newborn | NA | NA | NA | Vertex | Skull and dura absent | Y | Early | None | Integra® dermal regeneration template | Good | None | N |
| 10 | Seo DH, et al.^10^ | 2020 | Newborn | NA | NA | NA | Vertex | Skin and bone absent | N | Delayed | Not used | Autologous parietal bone graft + rotation flap | Good | None | N |
| 11 | Orgun D, et al.^11^ | 2017 | 3 days | Female | N | N | Vertex | Skin and bone absent | Y | Early | Moist dressing | Debridement and Type I collagen matrix | Good | Surgical site infection | N |
| 12 | Park ES, et al.^12^ | 2017 | Newborn | Male | N | N | Vertex | Skin and bone absent | N | Early | Acellular dermal matrix | None | Good | None | N |
| 13 | Lonie S, et al.^13^ | 2016 | Newborn | Female | N | N | Vertex | Skin and bone absent | N | Early | Mepilex Ag dressings | Cadaveric skin graft | Good | None | N |
| 14 | Rocha D, et al.^13^ | 2015 | Newborn | NA | N | N | Vertex | Skin and bone absent | N | Early | Acellular dermal matrix | None | Good | Bleeding | N |
| 15 | Frojd V, et al.^14^ | 2014 | Newborn | Male | N | N | Vertex | Skin and bone absent | N | Early | Aquacel Ag Hydrofiber | Debridement of the defect site | Good | None | N |
| 16 | Dutra LB, et al.^15^ | 2009 | Newborn | Female | N | N | Vertex | Skull and dura absent | Y | Delayed | Moist dressing initialy | Lypophylized dural substitute + bipedicle scalp reconstruction | Good | Cystic hydrocephalus | N |

**Supplementary Table 1. List of Reported Literatures about Aplasia Cutis Congenita of the Scalp**

**References**

1. Agyekum R, Darko K, Odame NYA, Gyebi EAA, Haizel EA, Kpoh FNA, Andani AHD. Surgical management of aplasia cutis congenita of the scalp and skull defect in a resource-limited setting: A case report. *Surg* *Neurol* *Int*. 2026;17:46.
2. Uribe-Cavero LJ, Chavez-Ecos FA, Chuman-Sanchez M, Parra-Huaroto A, Neyra-Leon J, Torres-Huamani DO, et al. Clinical Characteristics, Management, and Prognosis of Newborns with Aplasia Cutis Congenita Type I: A Systematic Review and Case Report. *Sage* *Open Pediatrics*. 2026;13. doi:[10.1177/30502225261444806](https://doi.org/10.1177/30502225261444806)
3. Pollock M, Leung R, Low NCK. A Novel Approach to Aplasia Cutis Congenita With PolyNovo BTM. *J Craniofac Surg*. 2025;36(3):e296-e297
4. Bouali S, Charfeddine SH, Ghedira K, Mechergui H, Abderrahmen K, Kallel J. Large aplasia cutis congenita of the vertex conservative management. *Childs Nerv Syst*. 2024;40(2):285-292.
5. Rose MD. An Extensive Case of Aplasia Cutis Congenita. *Cureus*. 2024;16(6): e63215.
6. AlMatrafi FR, Al Shammari AA, Al Nefily RM, AlAnazi RA, Abdulwahab AH, Ammar AS. Aplasia Cutis Congenita of the Scalp with Bone Defect and Exposed Sagittal Sinus in Trisomy 13 Newborn – a Case Report. *Front*. *Pediatr*. 2023;11:1142950.
7. Yang XF, Shi SW, Chen K. Case report: Recombinant human epidermal growth factor gel plus kangfuxin solution in the treatment of aplasia cutis congenita in a case with Adams–Oliver syndrome. *Front Surg*. 2023;9:107202.
8. Karakawa R, Yano T, Yoshimatsu H, Koto M, Nakao A, Ichi S. Use of Ultra-high-frequency Ultrasound for Aplasia Cutis Congenita of the Scalp. *Plast Reconstr Surg Glob Open*. 2021;9(10):e3876.
9. Scotti A, Benanti E, Augelli F, Baruffaldi Preis FW. A Case of Large Aplasia Cutis Congenita with Underlying Skull Defect: Effective Surgical Treatment with Integra® Dermal Regeneration Template. *Pediatr Neurosurg*. 2021;56(3):268-273.
10. Seo DH, Roh SG, Koh EJ, Shin JY, Chang SC, Lee NH. Reconstruction of Congenital Cranial Defect Using Autologous Bone Graft in Aplasia Cutis Congenita. *J Craniofac Surg*. 2020;31(3):e245-e247.
11. Orgun D, Horiguchi M, Hayashi A, Shimoji K, Arai H, Mizuno H. Conservative Treatment of Large Aplasia Cutis Congenita of the Scalp With Bone Defect With Basic Fibroblast Growth Factor Application. *J Craniofac Surg*. 2017;28(2):e154-e158.
12. Park ES, Park JH, Shin HS, Nam SM. Clinical Application of Acellular Dermal Matrix in the Treatment of Aplasia Cutis Congenita on Scalp. *J Craniofac Surg*. 2017;28(8):e788-e789.
13. Lonie S, Phua Y, Burge J. Technique for Management of Aplasia Cutis Congenita of the Scalp With a Skin Allograft. *J Craniofac Surg*. 2016;27(4):1049-1050.
14. Rocha D, Rodrigues J, Marques JS, Pinto R, Gomes A. Aplasia cutis congenita: a conservative approach of a case with large, extensive skin, and underlying skull defect. *Clin Case Rep*. 2015;3(10):841-844.
15. Fröjd V, Maltese G, Kölby L, Tarnow P. Conservative Healing of an 11 × 9-cm Aplasia Cutis Congenita of the Scalp with Bone Defect. *J Neurol Surg Rep*. 2014;75(02):e220-e223.
16. Dutra LB, Pereira MD, Kreniski TM, Zanon N, Cavalheiro S, Ferreira LM. Aplasia Cutis Congenita: Management of a Large Skull Defect With Acrania. *J Craniofac Surg*. 2009;20(4):1288-1292.
